# Health Equity After a Colorectal Cancer Screening Program

**DOI:** 10.1001/jamahealthforum.2026.1520

**Published:** 2026-06-12

**Authors:** Xuechen Xiong, Carmen S. Ng, Yikun Zhang, Jiayi Liang, Jo Yi Chow, Sharon S. L. Pang, Xinyu Du, Wai Lun Law, Wai Keung Leung, Gabriel M. Leung, Jianchao Quan

**Affiliations:** 1 School of Public Health, LKS Faculty of Medicine, The University of Hong Kong, Hong Kong SAR, China; 2 Department of Applied Social Sciences, The Hong Kong Polytechnic University, Hong Kong SAR, China; 3 Department of Surgery, School of Clinical Medicine, Li Ka Shing Faculty of Medicine, The University of Hong Kong, Hong Kong SAR, China; 4 Department of Medicine, School of Clinical Medicine, Li Ka Shing Faculty of Medicine, The University of Hong Kong

## Abstract

**Importance:**

Colorectal cancer (CRC) is the third most diagnosed cancer and the second leading cause of cancer death globally. Early identification of precancerous lesions and early-stage cancers can substantially reduce CRC incidence and improve survival. Hong Kong launched an organized colorectal cancer screening program (CRCSP) for the population in 2016.

**Objective:**

To assess the association of the CRCSP with the health equity of cancer screening participation among different socioeconomic groups over a 9-year period.

**Design, Setting, and Participants:**

Individual-level data from adults aged 50 to 75 years were analyzed from 3 repeated territory-wide population surveys in Hong Kong, China, between December 2014 to January 2022 to track trends in fecal test and colonoscopy participation. Disparities in screening uptake by socioeconomic status and income were assessed by concentration curves and indices. Survey-weighted generalized logistic regression models were used to assess the association between screening participation and socioeconomic factors. Data were analyzed from January 2025 to March 2026.

**Main Outcomes and Measures:**

Colorectal cancer screening utilization, specifically having ever undergone (1) a fecal test without symptoms and (2) a colonoscopy without symptoms.

**Results:**

This study analyzed 14 602 adults aged 50 to 75 years (mean [SD] age, 60.62 [7.03] years; 7716 female individuals [52.84%]). Participation in fecal testing increased from 19.88% (95% CI, 18.70-21.05) from 2014 to 2015 to 27.15% (95% CI, 26.12%-28.18%) from 2020 to 2022, and colonoscopy uptake rose from 17.65% (95% CI, 16.53%-18.77%) to 27.28% (95% CI, 26.24%-28.31%). Socioeconomic disparities narrowed after the CRCSP, with the concentration indices declining (fecal test participation, 0.11 from 2014-2015 to 0.02 from 2020-2022). Similar improvements were found for colonoscopy screening and across income groups. Screening participation was significantly associated with older age, higher education level, marital status, private housing, income, and socioeconomic status.

**Conclusions and Relevance:**

In this cross-sectional study, following the implementation of an organized population screening program, colorectal cancer screening participation increased and socioeconomic disparities narrowed over time. Overall uptake in Hong Kong remains low, and some disparities persist in younger age groups (age 50-59 years), single households, individuals with a primary and secondary school education, lower-income groups, and public housing residents. Efforts to increase participation are needed in certain groups to ensure health equity.

## Key Points

**Question:**

Did an organized population-wide colorectal cancer screening program in Hong Kong contribute to overall participation and equity across socioeconomic groups?

**Findings:**

In this cross-sectional study of 14 602 adults aged 50 to 75 years who participated in surveys, participation in fecal testing and colonoscopy screening increased by 36% to 55% from 2014 to 2022 following program implementation to reach more than 27% of the eligible population. Concentration indices showed socioeconomic inequalities narrowed considerably, but lower participation was associated with younger age, single households, lower incomes, and less education.

**Meaning:**

The study findings suggest that the population-wide availability of the screening program was associated with an increased overall uptake and narrowed disparities, but targeted efforts are needed for certain socioeconomic groups.

## Teaser

This cross-sectional study examines the association of the colorectal cancer screening program with the health equity of cancer screening participation among different socioeconomic groups.

## Introduction

Colorectal cancer (CRC) is the third most diagnosed cancer and the second leading cause of cancer death globally, with 1.9 million new cases and 0.9 million deaths in 2022.^1,2^ In Hong Kong, CRC is among the top 3 most common cancers and the second leading cause of cancer mortality,^3^ a pattern similar to other high-income East Asian settings. Early identification of precancerous lesions and early-stage cancers can substantially reduce CRC incidence and improve survival. Previous studies have indicated that implementing an organized screening program, such as fecal testing and colonoscopy, could reduce CRC mortality by 30% to 60% among appropriately screened populations.^4,5,6^ Organized CRC screening initiatives have been adopted in many countries to promote population-wide participation and reduce disparities among socioeconomic groups.^7^ Substantial socioeconomic inequalities persist, even in these mature programs.^8^ These experiences highlight the potential of an organized cancer screening program to improve overall access and the ongoing challenge in achieving equitable coverage for such programs.^9,10^

In Hong Kong and many other Asian settings, including mainland China, CRC screening uptake remains modest, with participation rates substantially lower than in Western countries.^11,12^ Studies in Chinese populations have identified common structural and behavioral barriers, including limited integration of preventive screening into routine primary care,^13^ reliance on opportunistic rather than invitation-based screening, cultural preferences for symptom-driven care, and lower public awareness of asymptomatic CRC screening.^13,14^ Socioeconomic gradients in health literacy, health care access, and affordability have further exacerbated inequalities in screening participation.^15^

Hong Kong launched its organized, population-wide colorectal cancer screening program (CRCSP) as a pilot in 2016, with full implementation in 2020.^16^ The program has been actively promoted since its inception through videos, printed materials, billboards, and television advertisements.^17^ Despite these efforts, participation rates remain relatively modest, with neither uptake of fecal testing nor colonoscopy exceeding 30% of eligible residents.^18,19^ It remains unclear whether the observed increases in screening uptake have been equitably distributed across socioeconomic groups. When overall screening participation is constrained by structural and socioeconomic barriers, gains in uptake may disproportionately accrue to more advantaged groups. Given the well-documented socioeconomic gradients in the CRC screening participation,^13,20^ this study aimed to assess the equity of CRC screening utilization among different socioeconomic groups following program implementation.

## Methods

### Setting

The Hong Kong CRCSP is a government-funded, population-wide initiative that heavily subsidizes CRC screening using a public-private partnership model. The program used a phased approach to expand eligibility in which it was piloted in 2016, regularized in 2018, and fully implemented in 2020. The scope was progressively widened from its initial pilot of residents aged 68 to 70 years in 2016 to aged 62 to 71 years in 2017, aged 61 to 75 years in 2018, aged 56 to 75 years in 2019, and finally to aged 50 to 75 years in 2020.^21,22^ Eligible participants were asymptomatic Hong Kong residents who had no symptoms suggestive of CRC, high-risk conditions, and recent CRC screening or examination.^23^

The primary screening test was the fecal immunohistochemistry test (FIT). The government subsidizes the costs of the first consultation with an enrolled primary care physician, participant pack (including 2 FIT tubes), and a second consultation if the FIT result is positive. The subsidy is $36 (HK$280) per consultation and a 1-off payment of $10 (HK$76) for enrollment.^24^ If the fecal test result is positive, a referral is made during the second consultation to an enrolled colonoscopy specialist for a colonoscopy examination (government subsidy of $998 [HK$7800] or $1088 [HK$8500] if polyp removal is necessary). Colonoscopy specialists may charge a copayment that does not exceed $128 (HK$1000) when providing the colonoscopy examination. Participants with a negative fecal test result received FIT rescreening every 2 years until age 75 years.^25^

### Study Design and Data

This study used a repeated cross-sectional survey design to assess the equity of CRC screening service utilization among different socioeconomic groups in Hong Kong. All data sources sampled exclusively from the Hong Kong population. We analyzed data from 3 large-scale, territory-wide population surveys of adults aged 50 to 75 years before and during the implementation of the CRCSP from 2014 to 2022. Data were sourced from population health surveys conducted by the Department of Health: the Population Health Surveys (PHS) from 2014 to 2015 and 2020 to 2022 and the Health Behavior Survey (HBS) from 2018 to 2019. The PHS captured the prelaunch and full-implementation periods, while the HBS captured the mid-implementation phase. All PHS and HBS surveys used a probability-based sampling of Hong Kong’s land-based, noninstitutional household population and collected harmonizable screening and socioeconomic measures to allow valid comparison of participation patterns over time (eTable 1 in Supplement 1).

Ethical approval was obtained from the institutional review board of the University of Hong Kong/Hospital Authority Hong Kong West Cluster. The analysis used anonymized secondary data, for which informed consent had been collected by the Department of Health during the original data collection; therefore, no additional consent was required for this secondary analysis. We followed the Strengthening the Reporting of Observational Studies in Epidemiology (STROBE) reporting guidelines.

### Outcomes

The main outcome was CRC screening utilization. Specifically, this was having ever undergone a fecal test without symptoms and a colonoscopy without symptoms.

### Statistical Analysis

Descriptive analyses were conducted to examine participation rates in CRC screening as stratified by age, sex, education level, marital status, housing type, household size, and personal income. To ensure representativeness, participation rates were weighted by age and sex, as reported by the Hong Kong Census and Statistics Department. Missing data were handled using a complete case analysis, as the proportion of missingness was minimal (<0.001%) and unlikely to introduce bias. We applied survey-weighted generalized logistic regression models to assess the association between the use of the CRC screening service and socioeconomic status. Results were reported as adjusted odds ratios (ORs).

We constructed our socioeconomic status (SES) index for people aged 50 to 75 years by applying a multifactor analysis on PHS 2020 to 2022 data. The index incorporated 3 domains: education level, housing type, and income. Factor loadings from the analysis were used as weights to calculate the composite SES score (eTable 2 in Supplement 1). A higher SES score reflected a higher socioeconomic position.

To further quantify and visualize socioeconomic disparities, we plotted concentration curves to illustrate the cumulative proportion of screening uptake against the cumulative proportion of participants ranked by SES. The deviation of these curves from the line of equality (the 45° diagonal) was used to evaluate inequality. To quantify the degree of inequality, we computed the concentration index (measured as twice the area between the concentration curve and line of equality), with higher values indicating greater disparities.^26,27^ Analyses were performed using R (version 4.3.3.; R Foundation).

### Sensitivity Analysis

We conducted sensitivity analyses to assess the robustness of our findings for alternative definitions of SES. First, we constructed an extended SES measure by adding 2 additional variables, household size and marital status, to our original multifactor SES index. We also applied the SDI6 social deprivation index developed by Wang et al^28^ for the general Hong Kong population (rather than specifically for adults aged 50-75 years). SDI6 is derived from 6 factors: education level, income, occupation, marital status, family composition, and household size.^28^ Finally, equity estimates based on these SES measures were compared with those obtained using income alone.

## Results

### CRC Screening Participation Rates

This study analyzed 14 602 adults aged 50 to 75 years (7716 female individuals [52.84%]). Across 3 survey waves (PHS 2014-2015, 4718 [32.3%]; PHS 2020-2022, 7506 [51.4%]; HBS, 2378 [16.3%]), the distribution of key demographic and socioeconomic characteristics were broadly comparable (Table 1). There was an overall upward trend in CRC screening utilization from 2014 to 2022. The weighted fecal test participation for the general population aged 50 to 75 years increased by 36.57% over the period, from 19.88% (95% CI, 18.70%-21.05%) from 2014 to 2015 to 27.15% (95% CI, 26.12%-28.18%) from 2020 to 2022. Colonoscopy uptake increased by 54.56% from 17.65% (95% CI, 16.53%-18.77%) from 2014 to 2015 to 27.28% (95% CI, 26.24%-28.31%) from 2020 to 2022.

**Table 1.** Characteristics of Survey Participants, From 2014 to 2015 and 2020 to 2022

Demographic variable	No. (%)			*P* value	SMD
	PHS, 2014-2015	HBS, 2018-2019	PHS, 2020-2022		
No.	4718	2378	7506	NA	NA
Age, y					
50-54	1314 (27.85)	577 (24.26)	1553 (20.69)	<.001	0.179
55-59	1152 (24.42)	626 (26.32)	1594 (21.24)		
60-64	975 (20.67)	499 (20.98)	1747 (23.27)		
65-69	711 (15.07)	413 (17.37)	1348 (17.96)		
70-75	566 (12.00)	263 (11.06)	1264 (16.84)		
Sex					
Female	2473 (52.42)	1233 (51.85)	4010 (53.42)	.31	0.021
Male	2245 (47.58)	1145 (48.15)	3496 (46.58)		
Education level^a^					
Primary	1635 (34.65)	NA	2018 (26.89)	<.001	0.169
Secondary	2479 (52.54)	NA	4394 (58.54)		
Tertiary	604 (12.80)	NA	1094 (14.58)		
Marital status^a^					
Married	3713 (78.70)	NA	5540 (73.81)	<.001	0.140
Single/never married	325 (6.89)	NA	747 (9.95)		
Divorced, separated, or widowed	680 (14.41)	NA	1206 (16.07)		
Housing					
Public	1676 (35.52)	827 (34.78)	2715 (36.17)	<.001	0.066
Subsidized	1006 (21.32)	566 (23.80)	1571 (20.93)		
Private	2036 (43.15)	980 (41.21)	3220 (42.90)		
Missing	0 (0.00)	5 (0. 21)	0 (0.00)		
Household size					
1	456 (9.67)	164 (6.90)	949 (12.64)	<.001	0.200
2	1241 (26.30)	626 (26.32)	2361 (31.45)		
3-5	2836 (60.11)	1435 (60.34)	3975 (52.96)		
≥6	185 (3.92)	153 (6.43)	221 (2.94)		
Personal income, $^a^					
≤1282	2838 (60.15)	NA	3961 (52.77)	<.001	0.197
1283-2564	1148 (24.33)	NA	1987 (26.47)		
2565-3846	328 (6.95)	NA	867 (11.55)		
≥3847	396 (8.39)	NA	691 (9.24)		
Missing	8 (0.17)	NA	0 (0.00)		
Employed^a^					
No	2417 (51.23)	NA	3954 (52.68)	.12	0.029
Yes	2301 (48.77)	NA	3552 (47.32)		

Abbreviations: HBS, Health Behavior Survey; NA, not applicable; PHS, Population Health Survey; SMD, standardized mean difference.

^a^ Pairwise comparison between PHS, 2014 to 2015, and PHS, 2020 to 2022, only due to a lack of comparable data in HBS, 2018 to 2019.

**Figure 1.**
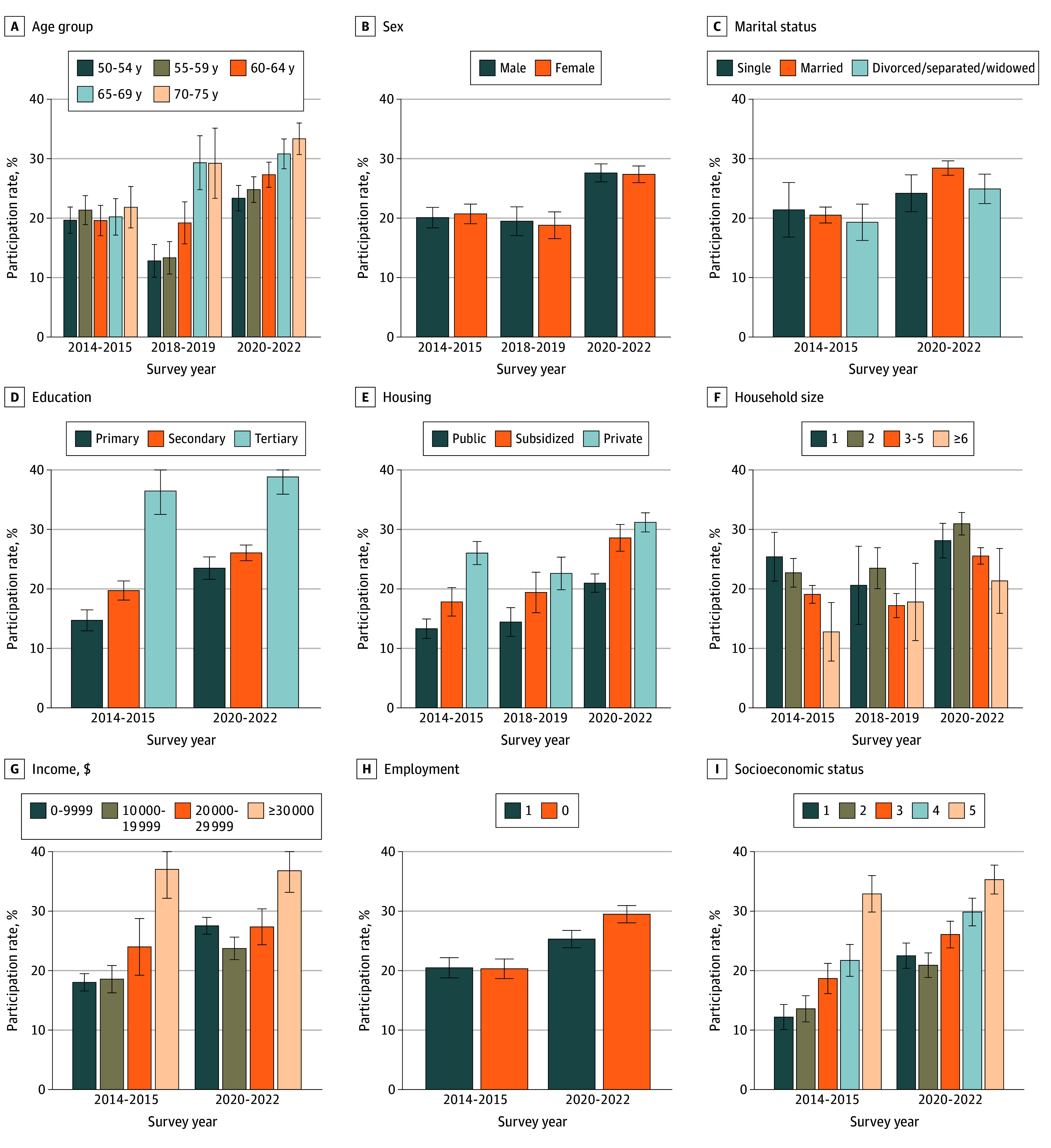
Bar Graphs of Participation in Fecal Test by Sociodemographic Group From 2014 to 2015 and 2020 to 2022 Sample-weighted proportions of fecal test and colonoscopy screening. Data were from the Population Health Survey from 2014 to 2015, Health Behavior Survey from 2018 to 2019, and Population Health Survey from 2020 to 2022.

Variations in CRC screening participation rates were observed among different demographic and socioeconomic groups across multiple survey periods (Figure 1; eFigure 1 in Supplement 1). Initially, from 2014 to 2015, fecal test uptake was relatively similar by age group, with rates ranging from 19.69% (95% CI, 17.47%-21.91%) in individuals aged 50 to 54 years to 21.88% (95% CI, 18.38%-25.37%) in 7 individuals aged 0 to 75 years. By 2020 to 2022, participation increased in all age groups, but particularly in older adults, reaching 23.39% (95% CI, 21.24%-25.55%) in individuals aged 50 to 54 years compared with 33.41% (95% CI, 30.75%-36.07%) in individuals aged 70 to 75 years. Differences by sex were minimal from 2014 to 2015 and 2020 to 2022, with female uptake increasing from 20.75% (95% CI, 19.10%-22.40%) to 27.42% (95% CI, 26.01%-28.83%), and male uptake from 20.12% (95% CI, 18.40%-21.84%) to 27.66% (95% CI, 26.14%-29.17%).

There were consistent disparities in screening participation by socioeconomic factors, such as education level, income, and housing. Individuals with a tertiary-level education consistently had higher participation rates (36.55% [95% CI, 32.64%-40.49%] in 2014-2015 and 38.94% [95% CI, 36.02%-41.86%] in 2020-2022) than those with a primary-level education (14.75% [95% CI, 12.99%-16.51%] and 23.55% [95% CI, 21.67%-25.44%], respectively). Similarly, higher-income groups had higher participation than lower-income groups. For example, individuals with monthly income of $3846 or more (HK$30 000) had a screening uptake of 37.13% (95% CI, 32.25%-42.00%) from 2014 to 2015 and 36.89% (95% CI, 33.24%-40.53%) from 2020 to 2022 compared with only 18.06% (95% CI, 16.60%-19.52%) and 27.61% (95% CI, 26.19%-29.03%) in those earning $1282 (HK$9999) or less. A monthly personal income of $1282 (HK$9999) or less generally corresponds to a low income based on the government’s poverty line.^29^ Likewise, participants residing in private housing had higher participation rates (26.09% [95% CI, 24.15%-28.03%] in 2014-2015 and 31.27% [95% CI, 29.66%-32.89%] in 2020-2022) than public housing residents (13.34% [95% CI, 11.69%-14.98%] and 21.02% [95% CI, 19.48%-22.56%], respectively). Although overall participation improved across all groups from 2014 to 2015 and 2020 to 2022, disparities between higher and lower socioeconomic groups persisted, albeit with modest narrowing of gaps over time (Figure 1; eFigure 2 in Supplement 1).

### Association Between Participation and SES

Table 2 shows the associations of CRC screening participation with demographic and socioeconomic factors across different survey periods. Age was associated with screening participation, with older age groups consistently showing a higher odds of undergoing fecal testing and colonoscopy compared with the reference group of individuals aged 50 to 54 years. Individuals aged 70 to 75 years exhibited the highest odds of screening participation (fecal test: OR, 2.16; 95% CI, 1.76-2.64; colonoscopy: OR, 2.04; 95% CI, 1.67-2.50; 2020-2022). Educational level was positively associated with screening uptake, with tertiary-educated individuals most likely to participate (fecal test: OR, 2.06; 95% CI, 1.70-2.50; colonoscopy: OR, 1.80; 95% CI, 1.48-2.18; 2020-2022). Single individuals had lower odds of participation compared with married individuals, although not all estimates reached statistical significance. Private housing and higher income were associated with increased screening participation. However, employment status was inversely associated with screening participation (employed individuals: fecal test OR, 0.83; 95% CI, 0.70-0.98; colonoscopy OR, 0.70; 95% CI, 0.59-0.83; 2020-2022). Most employed participants (5276 [90%]) were aged 50 to 64 years, representing the younger segment of the eligible age range.

**Table 2.** Association Between Colorectal Cancer Screening Utilization and Various Socioeconomic Indicators From 2014 to 2015 and 2020 to 2022

Characteristic	OR (95% CI)			
	Fecal test		Colonoscopy	
	PHS, 2014-2015	PHS, 2020-2022	PHS, 2014-2015	PHS, 2020-2022
Age, y				
50-54	1 [Reference]	1 [Reference]	1 [Reference]	1 [Reference]
55-59	1.21 (0.99-1.48)	1.16 (0.98-1.38)	1.19 (0.97-1.45)	1.27 (1.07-1.50)
60-64	1.19 (0.95-1.48)	1.46 (1.23-1.73)	1.37 (1.10-1.71)	1.64 (1.38-1.94)
65-69	1.35 (1.04-1.74)	1.81 (1.50-2.19)	1.71 (1.33-2.20)	1.95 (1.62-2.36)
70-75	1.50 (1.13-1.98)	2.16 (1.76-2.64)	1.30 (0.98-1.73)	2.04 (1.67-2.50)
Sex				
Female	1 [Reference]	1 [Reference]	1 [Reference]	1 [Reference]
Male	0.82 (0.70-0.97)	0.90 (0.80-1.00)	1.06 (0.90-1.24)	0.90 (0.81-1.00)
Education level				
Primary	1 [Reference]	1 [Reference]	1 [Reference]	1 [Reference]
Secondary	1.34 (1.12-1.60)	1.30 (1.14-1.48)	1.14 (0.96-1.35)	1.46 (1.28-1.66)
Tertiary	2.21 (1.71-2.86)	2.06 (1.70-2.50)	1.21 (0.93-1.58)	1.80 (1.48-2.18)
Marital status				
Married	1 [Reference]	1 [Reference]	1 [Reference]	1 [Reference]
Single/never married	0.80 (0.58-1.09)	0.76 (0.62-0.93)	0.75 (0.54-1.04)	0.76 (0.63-0.93)
Divorced, separated, or widowed	0.88 (0.70-1.11)	0.81 (0.69-0.95)	0.91 (0.73-1.14)	0.76 (0.65-0.89)
Housing				
Public	1 [Reference]	1 [Reference]	1 [Reference]	1 [Reference]
Subsidized	1.36 (1.10-1.67)	1.36 (1.17-1.57)	1.28 (1.05-1.57)	1.37 (1.19-1.58)
Private	1.62 (1.35-1.94)	1.32 (1.16-1.50)	1.63 (1.36-1.94)	1.34 (1.18-1.52)
Household size, No. of people				
1	1 [Reference]	1 [Reference]	1 [Reference]	1 [Reference]
2	0.77 (0.58-1.01)	0.96 (0.79-1.16)	0.92 (0.69-1.22)	0.95 (0.79-1.14)
3-5	0.67 (0.51-0.88)	0.80 (0.66-0.97)	0.78 (0.59-1.03)	0.78 (0.65-0.94)
≥6	0.45 (0.27-0.74)	0.62 (0.43-0.89)	0.64 (0.40-1.02)	0.56 (0.39-0.81)
Personal income, $				
≤1282	1 [Reference]	1 [Reference]	1 [Reference]	1 [Reference]
1283-2564	1.28 (1.02-1.62)	1.13 (0.95-1.35)	1.13 (0.90-1.43)	1.12 (0.94-1.34)
2565-3846	1.47 (1.06-2.03)	1.26 (1.01-1.57)	1.86 (1.36-2.56)	1.48 (1.19-1.84)
≥3847	2.06 (1.50-2.82)	1.59 (1.25-2.03)	2.21 (1.61-3.04)	2.18 (1.71-2.77)
Employment				
No	1 [Reference]	1 [Reference]	1 [Reference]	1 [Reference]
Yes	0.77 (0.62-0.95)	0.83 (0.70-0.98)	0.75 (0.61-0.93)	0.70 (0.59-0.83)

Abbreviations: OR, odds ratio; PHS, Population Health Survey.

**Figure 2.**
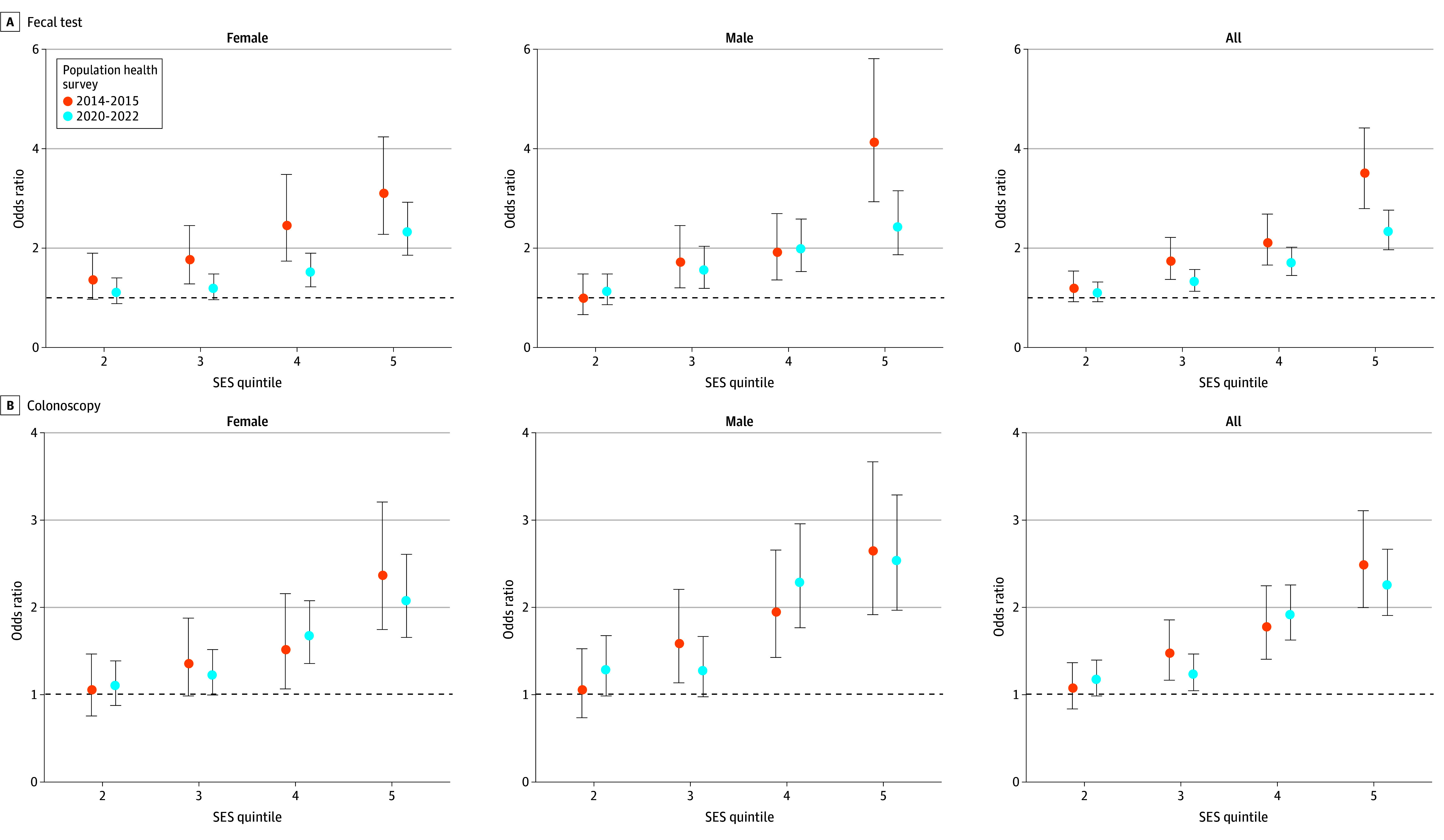
Box and Whisker Plots of the Association of Fecal Test and Colonoscopy Participation With Socioeconomic Status From 2014 to 2015 and 2020 to 2022 SES indicates socioeconomic status quintiles (1 = lowest, 5 = highest).

Following the CRCSP implementation, there were improvements in the equity of screening participation. Figure 2 and eFigures 3 and 4 in Supplement 1 show the socioeconomic disparities in CRC screening for fecal tests and colonoscopies and that these narrowed following the CRCSP implementation. Before the CRCSP, those with higher SES had significantly higher odds of screening (fecal test: OR, 3.52; 95% CI, 2.80-4.43; 2014-2015). After implementation of the CRCSP, there was greater convergence between SES groups; the odds ratio for higher SES groups decreased (fecal test: OR, 2.34; 95% CI, 1.97-2.77; 2020-2022), while the odds ratio for lower SES groups increased. Similar improvements in narrowing disparities were observed for colonoscopy uptake.

### Concentration Indices

**Figure 3.**
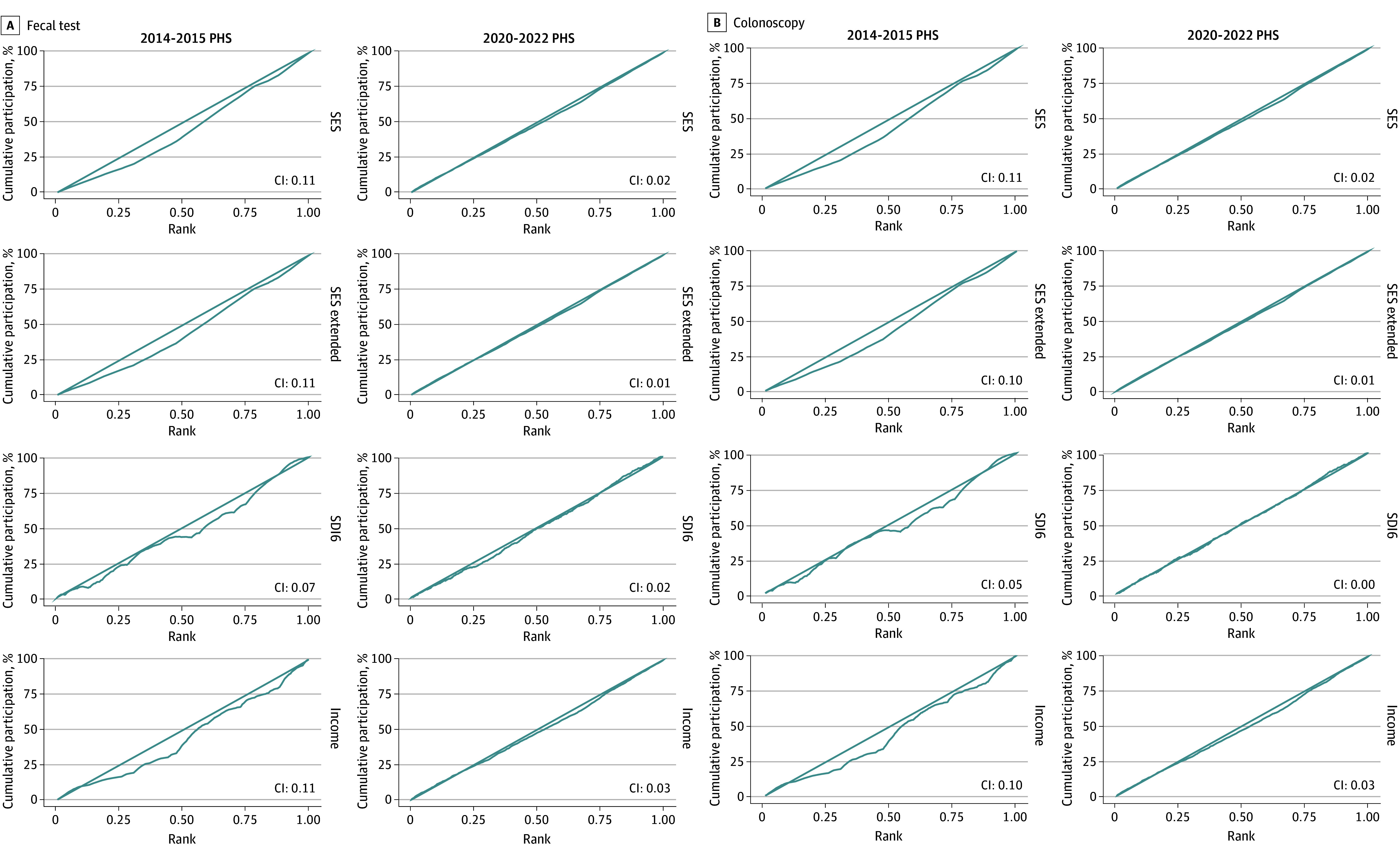
Line Graphs of Concentration Indices (CI) for Fecal Test Participation by Socioeconomic Status (SES) From 2014 to 2015 and 2020 to 2022 Positive value for CI indicates utilization is concentrated among higher SES groups, whereas a negative value indicates concentration among lower SES groups. We ranked SES and income from low to high. For SDI6, we reversed the rankings because higher scores indicate greater deprivation (which corresponds to lower SES) to facilitate consistent comparison. SES includes education, income, and housing type; SES extended includes these variables, as well as household size and marital status. SDI16 is a social deprivation index developed by Wang et al.^28^ PHS indicates population health survey.

The concentration indices indicated a reduction in socioeconomic inequalities for CRC screening participation over time. For fecal test participation, the concentration index was 0.11 (95% CI, 0.11-0.12) from 2014 to 2015, reflecting a distribution that favored higher SES, but this decreased to 0.02 (95% CI, 0.01-0.02) from 2020 to 2022, indicating a shift toward more equitable participation. Similarly, for colonoscopy participation, there was a reduction in the bias favoring higher SES, as the concentration index declined from 0.11 (95% CI, 0.10-0.11) from 2014 to 2015 and 0.02 (95% CI, 0.02-0.02) from 2020 to 2022. These trends were consistent for other measures of SES, such as personal income and SDI6, which also demonstrated decreasing concentration indices over the period (Figure 3).

## Discussion

To our knowledge, this cross-sectional study was the first to systematically assess equity effects before and after the implementation of an organized population CRC screening program in Hong Kong. Screening uptake increased steadily across demographic and socioeconomic groups following the rollout of the CRCSP. Participation in fecal tests increased from 19.88% of the eligible population in 2014 to 27.15% in 2022, and colonoscopy participation increased from 17.65% to 27.28%. Consistently higher participation rates were observed for older adults, married individuals, private housing residents, and those with a higher education level, income, or SES. Observed declines in concentration indices and narrower participation gaps over time suggest a temporal reduction in socioeconomic inequalities during the CRCSP implementation period.

These trends should be interpreted within the broader health policy context in Hong Kong. From 2014 to 2022, the government introduced several primary health care reforms aimed at strengthening prevention and early detection. Key initiatives included the establishment of district health centers^30^ starting in 2017 and the release of the Primary Healthcare Blueprint in 2022, which emphasized a shift toward prevention-focused, community-based care.^31^ Although these reforms were not specifically designed for CRC screening, they may have indirectly increased public awareness of preventive health services and encouraged greater engagement with primary care. Furthermore, the COVID-19 pandemic may have affected screening participation through disruptions to routine services and health care–seeking behavior, despite relatively limited community transmission in Hong Kong before 2022.

Although these trends indicate progress in health equity, participation in CRC screening in Hong Kong (46.2% of the eligible population in 2023) remains substantially lower than other high-income settings, where recent participation reached 68% to 74% in Finland, the US, and the UK.^8,32,33,34^ Beyond low overall uptake, we also observed persistent disparities in screening participation across demographic and socioeconomic groups. Individuals with lower educational attainment, lower income, or who were single or socially isolated consistently demonstrated lower participation rates. These socioeconomic gradients likely reflect broader structural and behavioral characteristics of Hong Kong’s mixed public-private health care system. Primary health care is largely delivered privately (75% of expenditures), highly fragmented, and heavily reliant on out-of-pocket payments (77% of private primary health care expenditures), which may present financial and access barriers for lower-income groups.^35^ Care coordination, such as preventive screening and gatekeeping, is not deeply integrated into routine primary care pathways, with only 23% of the population having a family physician and patients approaching through multiple points of contact in the health care process. Consultations are quick (typically less than 8 minutes) and episodic driven, with physician shopping behavior common (26% to 40% of patients consulting different physicians for the same illness episode).^36^ Local studies have shown that people with a lower health literacy may perceive low susceptibility to CRC or find screening procedures burdensome.^35,37^ Although CRCSP participants can mostly access the subsidized screening free of charge (more than 90% of primary care directory clinics and more than 70% of colonoscopy clinics do not charge any copayment for FIT or colonoscopy examination), the service model still requires individuals to proactively book a consultation with an enrolled private physician, which may disadvantage those with limited time, weaker navigation skills, or fewer social supports. Local social isolation may also reduce exposure to health information and peer encouragement.^37^ Together, these contextual factors help explain Hong Kong’s overall low participation rates and the persistent socioeconomic disparities observed, underscoring the need for targeted, equity-oriented efforts and system-level changes to primary and preventive care.

To address these gaps, tailored outreach strategies may be necessary. Experiences from overseas may offer practical insights, as regions with substantially higher CRC screening uptake often implement population-based invitation systems in which eligible individuals are centrally identified and mailed FIT kits or invitations at regular intervals.^38^ Finland’s biennial FIT program exemplifies such centralized coordination,^39^ while studies from the US show mailed FIT outreach, combined with patient navigation, was associated with improved screening uptake and follow-up colonoscopy in underserved populations.^40^ Adapting elements of these successful approaches, such as centralized invitations, mailed FIT kits, enhanced navigation support, and systematic follow-up, may promote more equitable screening participation in private and fragmented primary health care systems, such as Hong Kong.

### Strengths and Limitations

The study strengths lie in its large-scale, longitudinal, territory-wide datasets that encompassed several rounds of population health surveys, which provides insights into temporal changes in participation rates and disparities between socioeconomic groups. Several limitations should be acknowledged. First, we relied on multiple population-based surveys, as no single data source in Hong Kong provides longitudinally comparable and population-representative measures of CRC screening across all phases of the CRCSP rollout. Consequently, the analysis was based on repeated cross-sectional data rather than a longitudinal or quasiexperimental design. Thus, the findings reflect temporal associations rather than causal effects of the CRCSP, and the potential influence of other concurrent system-level or behavioral changes cannot be excluded. Second, all surveys used independent probability-based sampling frames, and no personal identifiers were available to permit linkage across datasets. Although repeated participation across waves cannot be entirely excluded, the likelihood is minimal, and any such occurrences would likely bias estimates toward the null. Third, the ever screened measure reflects lifetime participation and does not indicate when screening occurred or whether it was obtained through the CRCSP or privately. This limited our ability to assess recent screening behavior or program-specific uptake; therefore, the observed increases should be interpreted as broad population-level patterns rather than precise indicators of CRCSP participation. Finally, although the 2020 to 2022 survey period overlapped with the COVID-19 pandemic, Hong Kong experienced relatively limited transmission before the major Omicron surge in early 2022.^41^ However, pandemic-related disruptions, such as reduced service availability, changes in health care–seeking behavior, and temporary suspension of nonurgent care, were documented during this period and may have affected screening participation.^42^ Future postpandemic data will be essential to analyze long-term patterns.

## Conclusions

This cross-sectional study of health equity highlighted the progress and persistent challenges in achieving greater and more equitable participation. Socioeconomic disparities narrowed following the introduction of an organized, population-wide colorectal screening program, although some gaps remained for those who are younger, have lower educational attainment or lower income, are single, employed, or reside in public housing. There remains room for general improvement, as overall screening uptake remains lower than comparable high-income settings. Improvement strategies could include greater integration of cancer screening into primary care and expanding community outreach. Continued monitoring will be essential to ensure that all groups receive greater and more equitable benefits from cancer prevention efforts.

## Notes

Supplement 1. eTable 1. Information on territory-wide population health surveys eTable 2. Multifactor analysis for socioeconomic status eFigure 1. Participation in colonoscopy screening by socioeconomic status eFigure 2. Participation in fecal test and colonoscopy screening by socioeconomic status measures eFigure 3. Association of fecal test participation by age group and socioeconomic status quintile, 2014-15 to 2020-22 eFigure 4. Association of colonoscopy participation by age group and socioeconomic status quintile, 2014-15 to 2020-22

Supplement 2. Data sharing statement

## References

[1] International Agency for Research on Cancer. Cancer today. Accessed November 21, 2025. https://gco.iarc.who.int

[2] Sung H, Ferlay J, Siegel RL, . Global cancer statistics 2020: GLOBOCAN estimates of incidence and mortality worldwide for 36 cancers in 185 countries. CA Cancer J Clin. 2021;71(3):209-249. doi:10.3322/caac.21660 33538338

[3] Hong Kong Cancer Registry. Top ten cancers: hospital authority, the Hong Kong Special Administrative Region of the People’s Republic of China. Accessed November 21, 2025. https://www3.ha.org.hk/cancereg/topten.html

[4] Mandel JS, Church TR, Bond JH, . The effect of fecal occult-blood screening on the incidence of colorectal cancer. N Engl J Med. 2000;343(22):1603-1607. doi:10.1056/NEJM200011303432203 11096167

[5] Siegel RL, Miller KD, Jemal A. Cancer statistics, 2020. CA Cancer J Clin. 2020;70(1):7-30. 31912902 10.3322/caac.21590

[6] Mandel JS, Bond JH, Church TR, . Reducing mortality from colorectal cancer by screening for fecal occult blood: Minnesota colon cancer control study. N Engl J Med. 1993;328(19):1365-1371. doi:10.1056/NEJM199305133281901 8474513

[7] Zauber AG, Winawer SJ, O’Brien MJ, . Colonoscopic polypectomy and long-term prevention of colorectal-cancer deaths. N Engl J Med. 2012;366(8):687-696. doi:10.1056/NEJMoa1100370 22356322 PMC3322371

[8] Gov.UK. Bowel cancer screening annual report 2022 to 2023. Accessed November 21, 2025. https://www.gov.uk/government/publications/bowel-cancer-screening-annual-report-2022-to-2023/bowel-cancer-screening-standards-data-report-2022-23

[9] Chen Y, Zhang Y, Yan Y, . Global colorectal cancer screening programs and coverage rate estimation: an evidence synthesis. J Transl Med. 2025;23(1):811. doi:10.1186/s12967-025-06887-4 40696392 PMC12285180

[10] Pickwell-Smith BA, Spencer K, Sadeghi MH, Greenley S, Lind M, Macleod U. Where are the inequalities in colorectal cancer care in a country with universal healthcare? a systematic review and narrative synthesis. BMJ Open. 2024;14(1):e080467. doi:10.1136/bmjopen-2023-080467 38171631 PMC10773363

[11] Xia C, Basu P, Kramer BS, . Cancer screening in China: a steep road from evidence to implementation. Lancet Public Health. 2023;8(12):e996-e1005. doi:10.1016/S2468-2667(23)00186-X 38000379 PMC10665203

[12] Chen H, Li N, Ren J, ; Cancer Screening Program in Urban China. Participation and yield of a population-based colorectal cancer screening programme in China. Gut. 2019;68(8):1450-1457. doi:10.1136/gutjnl-2018-317124 30377193

[13] Chan DNS, Choi KC, Au DWH, So WKW. Identifying the factors promoting colorectal cancer screening uptake in Hong Kong using Andersen’s behavioural model of health services use. BMC Public Health. 2022;22(1):1228. doi:10.1186/s12889-022-13634-7 35725428 PMC9208701

[14] Huang RL, Liu Q, Wang YX, . Awareness, attitude and barriers of colorectal cancer screening among high-risk populations in China: a cross-sectional study. BMJ Open. 2021;11(7):e045168. doi:10.1136/bmjopen-2020-045168 34253663 PMC8276297

[15] Lin Y, Fan S, Chai W, . Barriers and facilitators to population participation in colorectal cancer screening: an umbrella review. BMC Health Serv Res. 2026;26(1):288. doi:10.1186/s12913-025-13879-z 41612391 PMC12924444

[16] Department of Health, the Government of the Hong Kong Special Administrative Region. Colorectal cancer screening programme: the government of the Hong Kong Special Administrative Region. Accessed November 21, 2025. https://www.colonscreen.gov.hk/en/public/index.html

[17] Centre for Health Protection, Department of Health, Government of the Hong Kong Special Administrative Region. March 2025: colorectal cancer screening and prevention. Accessed November 20, 2025. https://www.chp.gov.hk/files/pdf/ncd_watch_mar_2025_en.pdf

[18] Department of Health, Government of the Hong Kong Special Administrative Region. Report of population health survey 2020-22: the Government of the Hong Kong Special Administrative Region, the People’s Republic of China. Accessed November 20, 2025. https://www.chp.gov.hk/files/pdf/dh_phs_2020-22_part_1_report_eng_rectified.pdf

[19] Chan DNS, So WKW, Choi KC. Participation in a government-subsidised colorectal cancer screening programme for asymptomatic individuals in Hong Kong. Cancer Epidemiol. 2022;79:102174. doi:10.1016/j.canep.2022.102174 35533550

[20] Choi KC, So WK, Chen JM, Lau GC, Lee PC, Chan CW. Comparison study of uptake of colorectal cancer testing between ethnic minorities and the general population in Hong Kong. Asian Pac J Cancer Prev. 2015;16(17):7713-7720. doi:10.7314/APJCP.2015.16.17.7713 26625786

[21] Law CC, Wong CHN, Chong PSK, . Effectiveness of population-based colorectal cancer screening programme in down-staging. Cancer Epidemiol. 2022;79:102184. doi:10.1016/j.canep.2022.102184 35580366

[22] The Government of the Hong Kong Special Administrative Region. Eligibility of colorectal cancer screening programme. Accessed November 21, 2025. https://www.info.gov.hk/gia/general/202212/29/P2022122300554.htm

[23] Department of Health, Government of the Hong Kong Special Administrative Region. Who may enrol: the Government of the Hong Kong Special Administrative Region. Accessed November 21, 2025. https://www.colonscreen.gov.hk/en/public/programme/who_may_enrol.html

[24] The Government of the Hong Kong Special Administrative Region. Press releases: eligibility of colorectal cancer screening programme. Accessed March 9, 2026. https://www.info.gov.hk/gia/general/202312/28/P2023122700356.htm

[25] The Government of the Hong Kong Special Administrative Region. Press releases: DH to extend coverage of Colorectal Cancer Screening Programme to Hong Kong residents aged 50 to 75. Accessed March 9, 2026. https://www.info.gov.hk/gia/general/201912/19/P2019121800687.htm

[26] Wagstaff A, van Doorslaer E, Paci P. On the measurement of horizontal inequity in the delivery of health care. J Health Econ. 1991;10(2):169-205. doi:10.1016/0167-6296(91)90003-6 10113709

[27] Wagstaff A, van Doorslaer E. Equity in health care finance and delivery. Handbook of Health Economics. Elsevier; 2000:1803-1862.

[28] Wang K, Law CK, Zhao J, . Measuring health-related social deprivation in small areas: development of an index and examination of its association with cancer mortality. Int J Equity Health. 2021;20(1):216. doi:10.1186/s12939-021-01545-9 34579732 PMC8474923

[29] Census and Statistics Department, Government of the Hong Kong Special Administrative Region. Poverty situation: the Government of the Hong Kong Special Administrative Region. Accessed November 21, 2025. https://www.censtatd.gov.hk/en/scode461.html

[30] Health Bureau, Government of the Hong Kong Special Administrative Region. District Health Centre. Accessed November 21, 2025. https://www.dhc.gov.hk/en/index.html

[31] The Government of the Hong Kong Special Administrative Region. Press releases: government releases primary healthcare blueprint. Accessed March 9, 2026. https://www.info.gov.hk/gia/general/202212/19/P2022121900561.htm

[32] Eurostat. Cancer screening statistics. Accessed March 9, 2026. https://ec.europa.eu/eurostat/statistics-explained/index.php?title=Cancer_screening_statistics

[33] Ebner DW, Finney Rutten LJ, Miller-Wilson LA, . Trends in colorectal cancer screening from the National Health Interview Survey: analysis of the impact of different modalities on overall screening rates. Cancer Prev Res (Phila). 2024;17(6):275-280. doi:10.1158/1940-6207.CAPR-23-0443 38561018 PMC11148536

[34] US Centers for Disease Control and Prevention. Use of colorectal cancer screening tests. Accessed March 9, 2026. https://www.cdc.gov/colorectal-cancer/use-screening-tests/index.html

[35] Zhong CC, Xu W, Withers M, Wong MCS, Huang J. Patient perceptions and attitudes towards early onset colorectal cancer screening in Hong Kong: a cross-sectional study. Lancet Reg Health West Pac. 2025;55:101453. doi:10.1016/j.lanwpc.2024.101453

[36] Health Bureau, the Government of the Hong Kong Special Administrative Region. Primary healthcare blueprint supplement. Accessed November 21, 2025. https://www.primaryhealthcare.gov.hk/bp/en/supplementary-documents/challenges/

[37] Hu YH, Du Z, Cheung KW, . IDDF2025-ABS-0199 why did Hong Kong residents take colorectal cancer screening uptake or not? a focus group interview. Gut. 2025;74(suppl 3):A332. doi:10.1136/gutjnl-2025-IDDF.231

[38] Kava CM, Smith JL, Kobernik EK, . Interventions to increase colorectal cancer screening uptake in rural settings: a scoping review. Prev Chronic Dis. 2025;22:E44. doi:10.5888/pcd22.250025 40674655 PMC12335313

[39] Malila N, Anttila A, Hakama M. Colorectal cancer screening in Finland: details of the national screening programme implemented in Autumn 2004. J Med Screen. 2005;12(1):28-32. doi:10.1258/0969141053279095 15814016

[40] Dougherty M. Mailed fecal immunochemical tests and patient navigation to increase colon cancer screening in rural populations. JAMA Netw Open. 2025;8(3):e250939. doi:10.1001/jamanetworkopen.2025.0939 40094670

[41] Su Z, Zhang R, McDonnell D, . Sense and sensibility: pandemic lessons from Hong Kong. Disaster Med Public Health Prep. 2024;18(1):e239. doi:10.1017/dmp.2024.256 39473372

[42] Xin H, Wu P, Wong JY, . Hospitalizations and mortality during the first year of the COVID-19 pandemic in Hong Kong, China: an observational study. Lancet Reg Health West Pac. 2022;30:100645. 36438907 10.1016/j.lanwpc.2022.100645 PMC9682934

