# Supplementary material for: Health Equity After a Colorectal Cancer Screening Program
Source: JAMA Health Forum. 2026 Jun 12;7(6):e261520. doi: 10.1001/jamahealthforum.2026.1520 (PMC13263778; doi:10.1001/jamahealthforum.2026.1520)
Supplement: Supplement 1. — eTable 1. Information on territory-wide population health surveys eTable 2. Multifactor analysis for socioeconomic status eFigure 1. Participation in colonoscopy screening by socioeconomic status eFigure 2. Participation in fecal test and colonoscopy screening by socioeconomic status measures eFigure 3. Association of fecal test participation by age group and socioeconomic status quintile, 2014-15 to 2020-22 eFigure 4. Association of colonoscopy participation by age group and socioeconomic status quintile, 2014-15 to 2020-22 [file jamahealthforum-e261520-s001.pdf]

Supplemental Online Content

Xiong X, Ng CS, Zhang Y, et al. Health equity after a colorectal cancer screening program. *JAMA Health Forum*. 2026;7(6):e261520. doi:10.1001/jamahealthforum.2026.1520

eTable 1. Information on territory-wide population health surveys
eTable 2. Multifactor analysis for socioeconomic status
eFigure 1. Participation in colonoscopy screening by socioeconomic status
eFigure 2. Participation in fecal test and colonoscopy screening by socioeconomic status measures
eFigure 3. Association of fecal test participation by age group and socioeconomic status quintile, 2014-15 to 2020-22
eFigure 4. Association of colonoscopy participation by age group and socioeconomic status quintile, 2014-15 to 2020-22

This supplemental material has been provided by the authors to give readers additional information about their work.

© 2026 Xiong X et al. *JAMA Health Forum*.

eTable 1. Information on territory-wide population health surveys

Survey	Description
**Population Health Surveys (PHS)**	We analyzed data on participants aged 50-75 (n=4,718) in the Population Health Survey (PHS) 2014/15. PHS was a cross-sectional territory-wide population health survey conducted by the Department of Health from December 2014 to October 2015. It covers land-based, non-institutionalized populations in Hong Kong aged 15 and above, excluding foreign domestic helpers and visitors. Samples of replicates of living quarters used in PHS are selected by systematic replicated sampling from the Frame of Quarters maintained by Hong Kong's Census and Statistics Department. The Frame of Quarters contains records of all addresses of permanent quarters in built-up areas and records of area segments in non-built-up areas. Each replicate is a representative sample of a domestic household and each household member aged 15 and above is enumerated individually. We also analyzed data on participants aged 50-75 (n=7,506) in the PHS 2020-22 conducted between November 2020 and January 2022.
**Health Behaviour Survey (HBS)**	We analyzed data on participants aged 50-75 (n=2,378) in the Health Behaviour Survey (HBS) 2018/19. HBS was a population-based cross-sectional household survey conducted by the Department of Health. Like the PHS, the HBS targeted land-based, non-institutionalized populations aged 15 and above in Hong Kong, and recruited 5,903 individuals from 2,717 domestic households between April 2018 and February 2019. All eligible persons in the domestic households selected by systematic replicated sampling of residential quarters from the Frame of Quarters were invited to participate in the survey. The data was further adjusted by the differential response rates across the 18 District Council districts, and grossed-up to the control for the age and gender profile by types of housing of the participants.

© 2026 Xiong X et al. *JAMA Health Forum*.

eTable 2. Multifactor analysis for socioeconomic status

Variables	Factor	Factor	Factor
Education	0.585	0.618	0.617
Income	0.564	0.588	0.589
Housing type	0.498	0.522	0.522
Household size	0.062	-0.024	--
Marital status		--	--
Married	-0.472	--	--
Single/Never	1.935	--	--
Divorced/Separated/Widowed	0.960	--	--

© 2026 Xiong X et al. *JAMA Health Forum*.

eFigure 1. Participation in colonoscopy screening by socioeconomic status

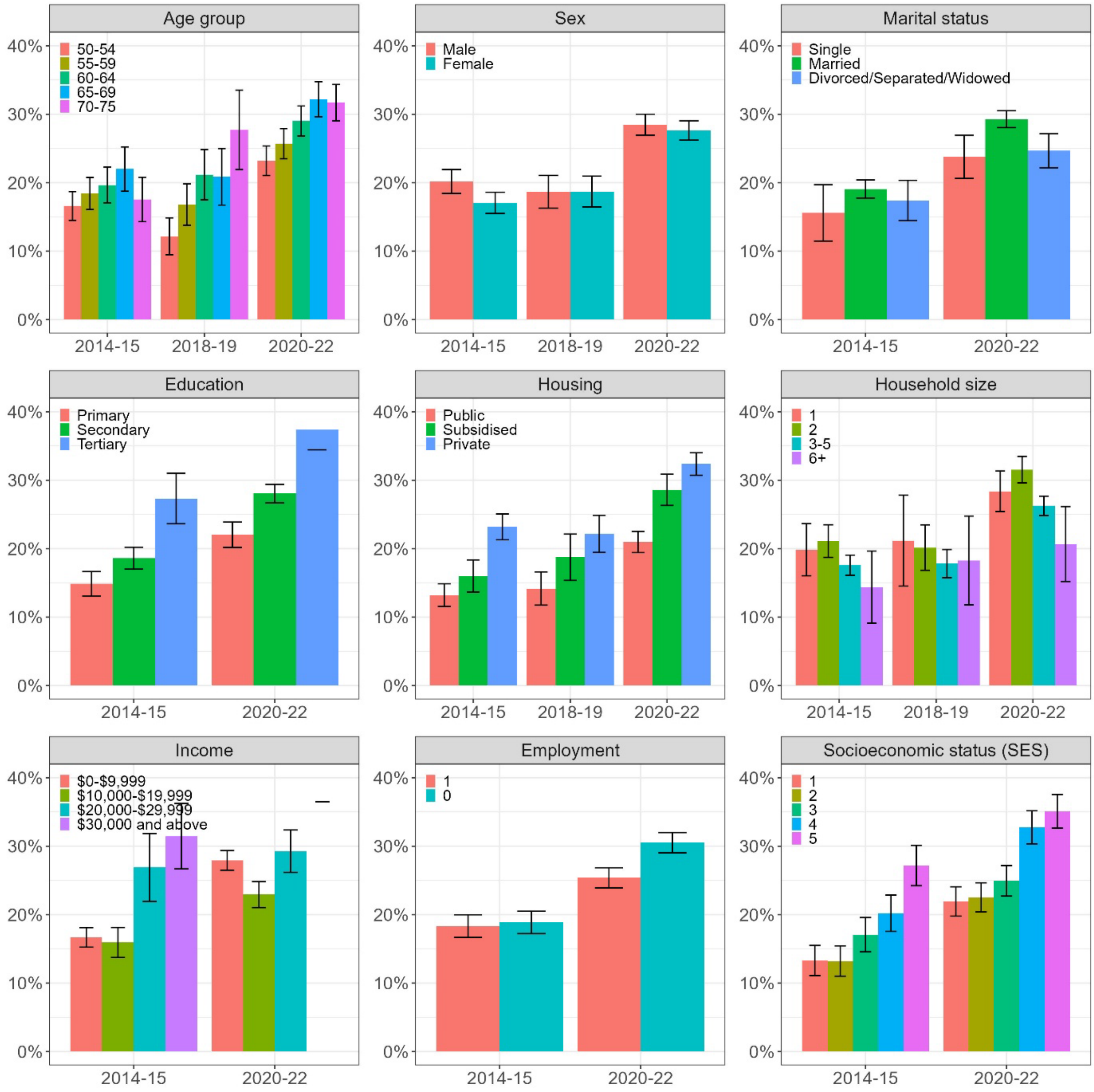

Sample weighted proportions of fecal test and colonoscopy screening.
Data: Population Health Survey 2014-15, Health Behaviour Survey 2018-19, and Population Health Survey 2020-22.

© 2026 Xiong X et al. *JAMA Health Forum*.

eFigure 2. Participation in fecal test and colonoscopy screening by socioeconomic status measures

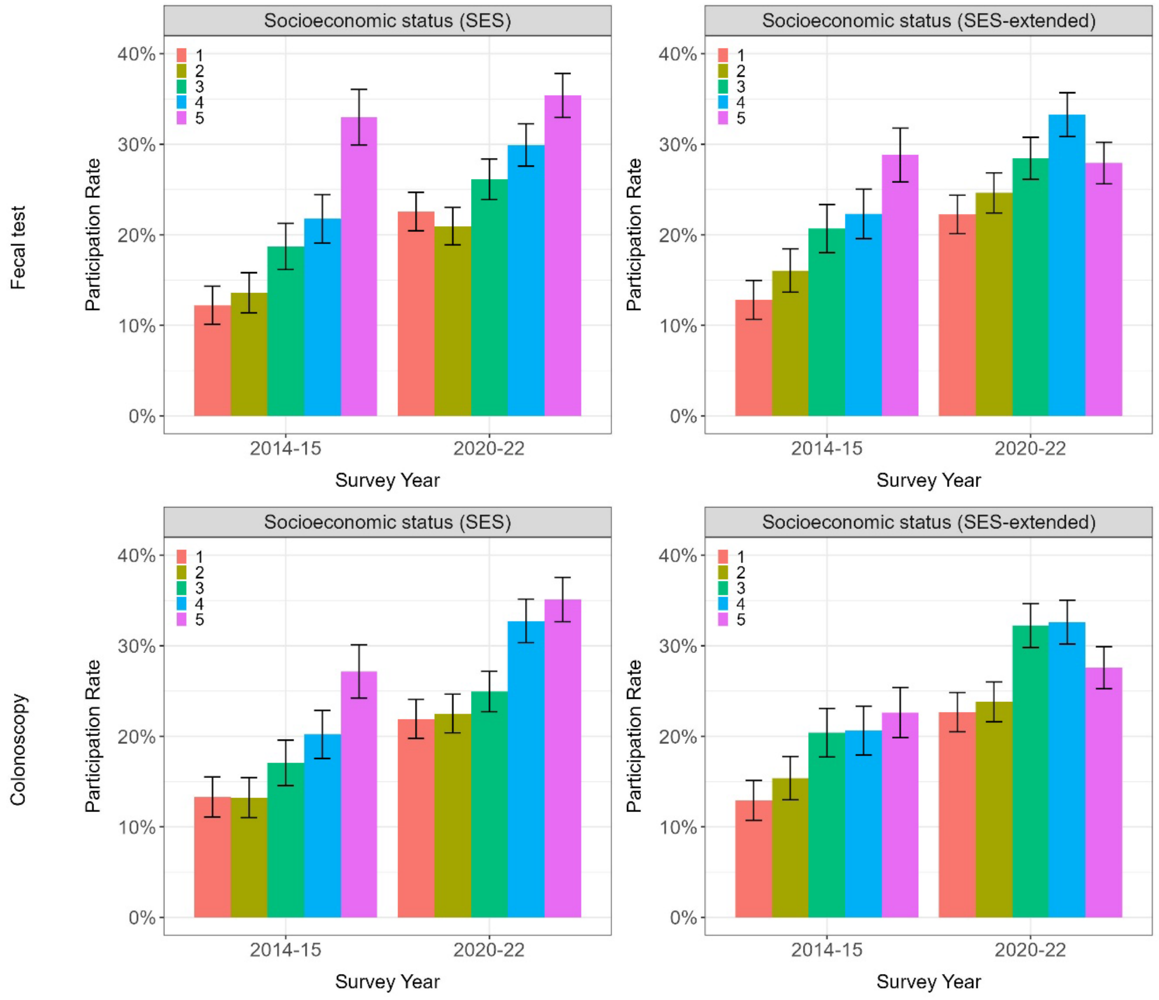

SES: socioeconomic status; SES includes education, income, and housing type; SES-extended includes these variables as well as household size and marital status

© 2026 Xiong X et al. *JAMA Health Forum*.

eFigure 3. Association of fecal test participation by age group and socioeconomic status quintile, 2014–15 to 2020–22

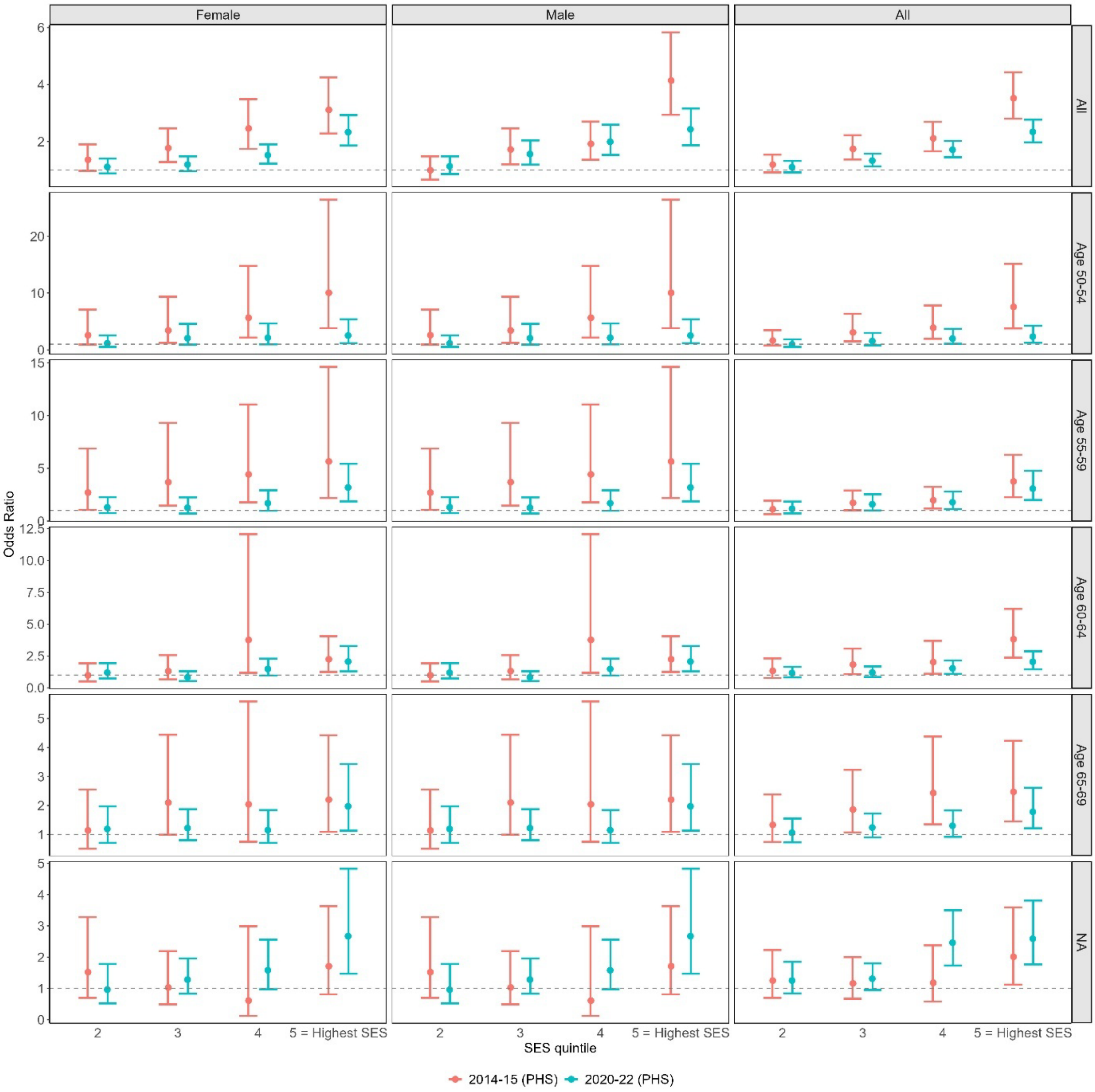

PHS, Population health survey; SES, socioeconomic status quintile (1 = lowest, 5 = highest).

© 2026 Xiong X et al. *JAMA Health Forum*.

eFigure 4. Association of colonoscopy participation by age group and socioeconomic status quintile, 2014-15 to 2020-22

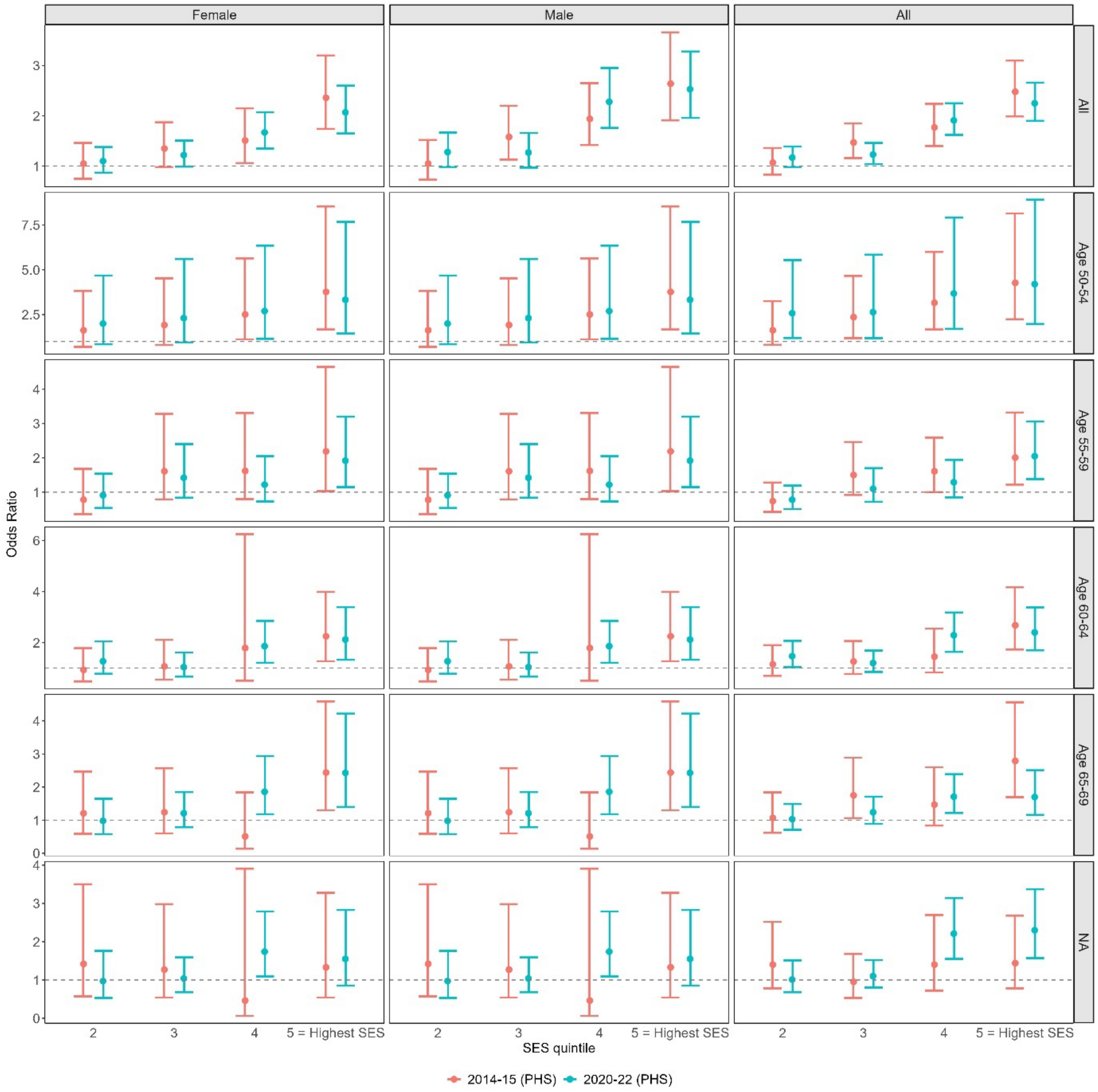

PHS, Population health survey; SES, socioeconomic status quintile (1 = lowest, 5 = highest).

© 2026 Xiong X et al. *JAMA Health Forum*.
