# Supplementary material for: Health Equity After a Colorectal Cancer Screening Program
Source: JAMA Health Forum. 2026 Jun 12;7(6):e261520. doi: 10.1001/jamahealthforum.2026.1520 (PMC13263778; doi:10.1001/jamahealthforum.2026.1520)
Supplement: Supplement 2. — Data sharing statement [file jamahealthforum-e261520-s002.pdf]

Data Sharing Statement

Xiong. Health Equity After a Colorectal Cancer Screening Program. *JAMA Health Forum*. Published June 12, 2026. doi:10.1001/jamahealthforum.2026.1520

Data
Data available: No

Additional Information
Explanation for why data not available: The data used in this study are not publicly available as they are owned by the Hong Kong Department of Health and subject to data-use restrictions that prohibit external sharing.
